# Underreporting of Human Alveolar Echinococcosis, Germany

**DOI:** 10.3201/eid1406.071173

**Published:** 2008-06

**Authors:** Pernille Jorgensen, Matthias an der Heiden, Petra Kern, Irene Schöneberg, Gérard Krause, Katharina Alpers

**Affiliations:** * Robert Koch Institute, Berlin, Germany; † European Echinococcosis Registry, Ulm, Germany

**Keywords:** Human alveolar echinococcosis, capture-recapture, surveillance, dispatch

## Abstract

We estimated the total number of human alveolar echinococcosis cases in Germany from 2003 through 2005 using the multiple source capture-recapture method. We found a 3-fold higher incidence of the disease than that shown by national surveillance data. We propose a revision of the reporting system to increase case ascertainment.

## Abstract 2

Underreporting of Human Alveolar Echinococcosis, Germany

Human alveolar echinococcosis (AE), caused by the metacestode stage of the fox tapeworm *Echinococcus multilocularis,* is a rare zoonosis in Germany, mainly occurring in the south (*1*). The parasite predominantly develops in the liver of the human host, where the infiltrating growth can cause serious damage (*2*). Untreated AE has a very high fatality rate (*3*); when the patient survives, the cost of life-long treatment is substantial, projected at US $300,000 per patient (*4*). *E. multilocularis* infection is highly endemic among foxes in Germany, and studies indicate that the parasite’s geographic range has widened in recent years (*5*,*6*). Growing fox populations in Europe, especially in urban zones, have drawn attention to a potential increased risk for humans (*7*–*9*).

In 2001, AE reporting became mandatory in Germany (*10*). Diagnosis of echinococcosis by serologic testing and histopathologic examination is reportable to the Robert Koch Institute (RKI) by microbiologic laboratories and pathologists, respectively. However, reports rarely come from pathologists. Referring physicians must provide additional diagnostic data (e.g., imaging findings and clinical information to confirm serologically diagnosed AE) but are not required to report cases independent of the laboratory diagnosis. Additionally, clinicians voluntarily report AE case-patients with active lesions to the European Echinococcosis Registry (EER) associated with the clinical referral center for AE in Ulm, Germany (*11*).

We conducted a 3-source capture–recapture analysis to generate an estimate of the true number of AE cases in Germany from 2003 through 2005. On the basis of this estimate, we assessed the sensitivity of the national surveillance system.

## The Study

The capture–recapture method estimates unascertained cases by comparing data from >2 different sources. It requires that persons have a correct diagnosis and equal probability of inclusion (catchability) and that the study population be closed. If only 2 sources are used, these should be independent (*12*).

We used 3 data sources: RKI, EER, and a pathologists’ survey (PAS) conducted in June 2006 among all registered pathology laboratories in Germany (≈525). Pathologists were requested to complete a questionnaire reporting all echinococcosis cases diagnosed from 2003 through 2005 to RKI.

We defined confirmed AE case-patients as persons with positive results of histopathologic examination or with liver lesion showing typical morphologic features, identified by imaging techniques. Only case-patients with a first diagnosis from 2003 through 2005 were included. Because reporting of AE is anonymous, we used 3 proxy matching identifiers. Matching criteria were identical: 1) year and month of birth, 2) sex, and 3) year and month of diagnosis (±6 months to allow for time variability of different diagnostic methods). For case-patients for whom month of birth or month of diagnosis was missing in >1 source, the first 3 digits of the case-patient’s postal code or the referring physician’s postal code had to be identical in addition to the above criteria.

The distribution of matched and unmatched observed cases by source is displayed in a Venn diagram (Figure). To predict the frequency of unascertained cases, we constructed log-linear models. Each model included a variable for each source and up to 3 possible interaction variables between sources. The saturated model included all 3 interactions, whereas the independent model assumed no interactions (*13*).

**Figure:**
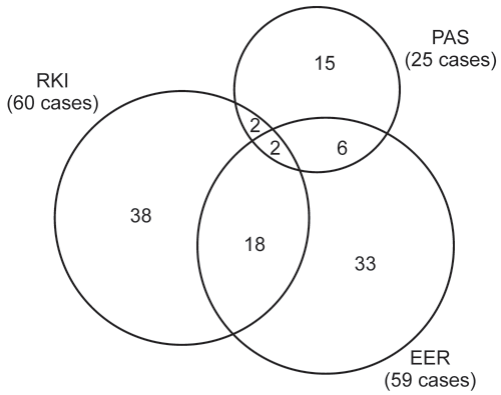
Venn diagram illustrating the distribution of confirmed first-diagnosis human alveolar echinococcosis cases from 2003 through 2005 in Germany by source and number of matches between sources. Data as of March 2007. RKI, Robert Koch Institute; EER, European Echinococcosis Registry; PAS, pathologists’ survey.

We selected the final model using Akaike’s Information Criterion (AIC), which indicates how well a model fits the data, considering the number of variables included. Small values of AIC correspond to a better adapted model (*12*). Ninety-five percent goodness-of-fit confidence intervals (95% CI) were calculated based on the likelihood ratio, to allow asymmetric intervals and avoid underestimation of the upper and lower limits (*14*).

The sensitivity of RKI data was estimated by dividing the number of cases reported to RKI by the total number of cases (*N*) from the selected model. Analysis was performed with Stata 9.0 (StataCorp, College Station, TX, USA) (*15*).

A total of 60 confirmed cases were reported to RKI; EER registered 59. The response rate for PAS was 64% (335 of 525 surveyed). Pathologists reported 49 AE cases in the survey, of which 25 were the first diagnosis, 5 were previously diagnosed, and 19 had no date of first diagnosis. Table 1 summarizes case-patient characteristics by source. From 2003 through 2005, 114 confirmed cases were recorded by the combined sources, of which 28 could be matched (Figure).

**Table 1:** Main characteristics of human alveolar echinococcosis case-patients by source, Germany, 2003–2005*

Characteristic	RKI (n = 60)	EER (n = 59)	PAS (n = 25)
Median age, y (range)	52 (15–92)	53 (17–81)	52 (18–81)
Female sex, no. (%)	32 (53)	29 (49)	14 (56)
Residence south Germany, no. (%)†	35 (76)‡	46 (77)	10 (53)§

*RKI, Robert Koch Institute; EER, European Echinococcosis Registry; PAS, pathologists’ survey. Data as of March 2007. †Case-patients for whom the 3 first digits of the residential postal code was >600. ‡Data available for 46 case-patients. §Data available for 19 case-patients.

Log-linear estimates for *N* ranged from 184 to 399 cases (Table 2). Model 5, with the single interaction term between RKI and PAS, was selected as the best fitting (AIC = ­–3.33) model. According to this model, 70 cases were missed, yielding 184 cases (95% CI 150–242) over 3 years. This corresponds to 61 cases (95% CI 50–81) annually, with an incidence rate of 0.07/100,000 persons. The lower estimate in model 5, compared with that of the independent model, suggested a negative dependence between RKI and PAS reports. Sensitivity of RKI was 33% (95% CI 25%–40%).

**Table 2:** Log linear estimates of the total number of alveolar echinococcosis cases, Germany, 2003–2005*

Models†	df	AIC	*x*	*N*	95% CI for *N*
Saturated: Interaction (RKI, EER) and (RKI, PAS) and (EER, PAS)	0	0.00	174	288	129–2020
Interaction (RKI, EER) and (RKI, PAS)	1	–1.49	83	197	143–358
Interaction (RKI, PAS) and (EER, PAS)	1	–1.33	70	184	148–253
Interaction (RKI, EER) and (EER, PAS)	1	–1.66	285	399	189–1961
Interaction (RKI, PAS)	2	–3.33	70	184	150–242
Interaction (RKI, EER)	2	–1.57	134	248	171–430
Interaction (EER, PAS)	2	–0.08	93	207	163–287
Independent (no interactions)	3	–1.94	89	203	163–268

*RKI, Robert Koch Institute; EER, European Echinococcosis Registry; PAS, pathologists’ survey; df, degrees of freedom; AIC, Akaike Information Criterion (measures how well the model fits the data [small values indicate a better fit]); *x*, estimate of unascertained cases; *N*, estimate of total number of cases (total number of observed cases + *x*); CI, goodness-of-fit–based confidence interval. †Each model includes all first-order terms. The first model (saturated) adjusts for dependencies between all 3 source pairs; the second model adjusts for possible dependencies between RKI and EER, and between RKI and PAS, etc.

## Conclusions

We estimated that the national surveillance system failed to detect 67% of AE cases in Germany over 3 years. Underreporting may occur for several reasons. Pathologists might be unaware of their obligation to report. Furthermore, reports almost exclusively come from microbiologic laboratories, and, consequently, case-patients who do not undergo serologic testing, or who have seronegative results, are likely to be missed. Finally, the reporting procedure is arduous because forms are detailed and must be first ordered from RKI.

Capture–recapture estimates can be biased if the underlying assumptions are violated. Because case identification was based on several variables, the potential for mismatching was considered small. However, the lenient criteria may have led to overmatching. Including more or fewer matching criteria had only a small effect on the estimate.

In the final models, we excluded cases reported through PAS when first-diagnosis status was unknown. Log-linear analysis that included these cases resulted in a higher estimate; therefore, we are confident that the exclusion did not overestimate the number of cases. Varying catchability can be addressed by stratification. Although the sources differed with regard to geographic distribution, we considered stratified analysis inappropriate due to missing postal codes for several case-patients, zero values in 1 stratum, and small numbers in general, which would increase the uncertainty around our estimate.

AE is not equally distributed in Germany, and the different geographic distribution of cases reported by PAS compared with RKI and EER indicated that PAS had missed case-patients mainly from the south. The number of histopathologically diagnosed cases was therefore likely underestimated. The importance of this for the estimated true number of AE case-patients presented here cannot be ascertained.

The negative dependence between RKI and PAS can be explained by diagnostic practices. Unpublished data from EER suggest that histopathologically diagnosed cases are less likely to have serologic test results than those without histopathologic examination. If a case-patient has had a histopathologic examination with positive results early in the diagnostic decision-making process, additional serologic testing is unnecessary, which reduces the chance of these case-patients being reported to RKI. Reporting to EER is independent of serologic testing, which could explain the greater overlap between EER and PAS than between RKI and PAS.

Despite the limitations, the study did demonstrate poor reporting of AE. To improve the national surveillance system, the focus of reporting should be shifted from microbiologic laboratories and pathologists to referring physicians, who usually collate the various diagnostic results.

Sustaining a surveillance system for AE in Germany is a major challenge because the disease is rare. However, a recent report on increasing human AE in neighboring Switzerland (*8*) underlines the importance of an effective surveillance system with adequate sensitivity to detect changes in disease incidence in order to guide strategies for prevention and control.
